# Remarks on Chilblain

**Published:** 1838-02-28

**Authors:** W. S. W. Ruschenberger

**Affiliations:** Surgeon U. S. Navy, &c.; Philadelphia


*Remarks on Chilblain, by* W. S. W. Ruschenberger, M. D., *Surgeon U. S. Navy, &c.*

The term pernio, kibe, or chilblain, is applied to designate a peculiar condition of the soft parts, which differs in the kind of pain and in other particulars from ordinary inflammation. In the milder cases, there is scarcely any visible change in the part from its normal condition, but there is an itching pain which sometimes deprives the patient of sleep. To relieve it, he exposes the affected part to the air or immerses it in cold water, which assuages the itching, at least during the treatment. When the disease is more active, the part becomes mottled or dark coloured, and swells; the skin cracks or chaps, and ulceration takes place, which sometimes results very seriously.

Chilblains are most commonly seated in parts remote from the heart, where the circulation is neither active nor vigorous, as on the instep, the heel, the toes, the back of the hand, nose, ears, &c. They are caused by suddenly changing the temperature several degrees; a part exposed to a moderate degree of cold, say 32° to 35°F. for some time, and then suddenly to a temperature considerably more elevated, say 75°F. would probably suffer from chilblain, but if the change be gradually brought about, no such phenomenon would take place. Long continued exposure to a moderate degree of cold, particularly if accompanied by moisture, will also cause chilblain. I have known the greater part of the crew of a sloop of war to be attacked with chilblains, from exposure to rainy, sleety weather for eight or ten days; but I do not recollect a case being reported until the temperature began to rise. A similar observation is made by Baron Larrey.

Intense cold causes another form of disease, denominated frostbite, which frequently occasions a loss of the toes, and sometimes even of the fingers.

In September, 1831, off Cape Horn, the crew of the U. S. Ship Falmouth suffered severely and generally from chilblains; and again, in the same region, in November, 1833; also a few cases occurred on board the U. S. Ship Peacock, off Cape Horn, is August, 1837. All those cases, where ulceration had not taken place, were entirely relieved by smearing the part with balsam copaibæ once or twice, and very few required more than a third application of the remedy.

*Philadelphia, Feb.* 20, 1838.

